# Evolution of DNA polymerases: an inactivated polymerase-exonuclease module in Pol ε and a chimeric origin of eukaryotic polymerases from two classes of archaeal ancestors

**DOI:** 10.1186/1745-6150-4-11

**Published:** 2009-03-18

**Authors:** Tahir H Tahirov, Kira S Makarova, Igor B Rogozin, Youri I Pavlov, Eugene V Koonin

**Affiliations:** 1 Eppley Institute for Research in Cancer and Allied Diseases, University of Nebraska Medical Center, Omaha, Nebraska 68198-7696, USA; 2 National Center for Biotechnology Information, National Library of Medicine, National Institutes of Health, Bethesda, MD 20894, USA

## Abstract

**Background:**

Evolution of DNA polymerases, the key enzymes of DNA replication and repair, is central to any reconstruction of the history of cellular life. However, the details of the evolutionary relationships between DNA polymerases of archaea and eukaryotes remain unresolved.

**Results:**

We performed a comparative analysis of archaeal, eukaryotic, and bacterial B-family DNA polymerases, which are the main replicative polymerases in archaea and eukaryotes, combined with an analysis of domain architectures. Surprisingly, we found that eukaryotic Polymerase ε consists of two tandem exonuclease-polymerase modules, the active N-terminal module and a C-terminal module in which both enzymatic domains are inactivated. The two modules are only distantly related to each other, an observation that suggests the possibility that Pol ε evolved as a result of insertion and subsequent inactivation of a distinct polymerase, possibly, of bacterial descent, upstream of the C-terminal Zn-fingers, rather than by tandem duplication. The presence of an inactivated exonuclease-polymerase module in Pol ε parallels a similar inactivation of both enzymatic domains in a distinct family of archaeal B-family polymerases. The results of phylogenetic analysis indicate that eukaryotic B-family polymerases, most likely, originate from two distantly related archaeal B-family polymerases, one form giving rise to Pol ε, and the other one to the common ancestor of Pol α, Pol δ, and Pol ζ. The C-terminal Zn-fingers that are present in all eukaryotic B-family polymerases, unexpectedly, are homologous to the Zn-finger of archaeal D-family DNA polymerases that are otherwise unrelated to the B family. The Zn-finger of Polε shows a markedly greater similarity to the counterpart in archaeal PolD than the Zn-fingers of other eukaryotic B-family polymerases.

**Conclusion:**

Evolution of eukaryotic DNA polymerases seems to have involved previously unnoticed complex events. We hypothesize that the archaeal ancestor of eukaryotes encoded three DNA polymerases, namely, two distinct B-family polymerases and a D-family polymerase all of which contributed to the evolution of the eukaryotic replication machinery. The Zn-finger might have been acquired from PolD by the B-family form that gave rise to Pol ε prior to or in the course of eukaryogenesis, and subsequently, was captured by the ancestor of the other B-family eukaryotic polymerases. The inactivated polymerase-exonuclease module of Pol ε might have evolved by fusion with a distinct polymerase, rather than by duplication of the active module of Pol ε, and is likely to play an important role in the assembly of eukaryotic replication and repair complexes.

**Reviewers:**

This article was reviewed by Patrick Forterre, Arcady Mushegian, and Chris Ponting. For the full reviews, please go to the Reviewers' Reports section.

## Background

DNA-dependent DNA polymerases (DdDps) are essential components of all cellular life forms inasmuch as genomes of all modern cells consist of DNA whose replication requires the activity of one or more DdDps [1,2]. Most of the DNA viruses with relatively large genomes also encode their own DdDps [3]. The great majority of cellular organisms possess several DdDps that operate during DNA chain elongation during replication and/or in diverse repair processes [4,5].

Structural and inferred evolutionary relationships between DdDps comprise a complex network. There are several families of DdDps that are only distantly related or unrelated to each other [6]. The replicative polymerases are sharply divided between the bacterial and archaeal-eukaryotic types that appear not to be homologous [7,8]. In bacteria, replication is performed by C-family polymerases that are not found in archaea or eukaryotes, whereas all archaea and eukaryotes, as well as a huge diversity of viruses, encode B-family polymerases that are responsible for genome replication in all eukaryotes and some of the archaea [6,9]. All eukaryotes, in particular, possess four paralogous B-family polymerases denoted Pol α, Pol δ, Pol ε, and Pol ζ involved in DNA replication and repair [5,10]. Of these, Pol α and Pol δ are essential components of the DNA replication machinery; Pol ε has an apparent role in replication, but its exact function is less clear, whereas Polζ is involved in translesion DNA synthesis [11-17]. Euryarchaeota, in addition, possess a distinct D family polymerase that seems to make a substantial contribution to replication (the replication of archaeal DNA is not understood in as much detail as bacterial or eukaryotic replication) and is unrelated to both B and C family polymerases [18-20]. Recently, PolD was detected also in the putative phyla Nanoarchaeota [21], Thaumarchaeota (formerly mesophilic Crenarchaeota) [22], and Korarchaeota [23], suggesting the possibility that this DdDp is ancestral in archaea.

Here we report results of comparisons of protein sequences of eukaryotic and archaeal DdDps that reveal unexpected aspects of their domain architectures and evolution, and lead to specific functional implications.

## Results and Discussion

### Inactivated polymerase and exonuclease domains in the C-terminal portion of Pol ε

Pol ε, one of the paralogous B family polymerases that are conserved in all eukaryotes, is a very large protein that typically consists of 2000 or more amino acid residues [17]. The functionally characterized proofreading 3'-5' exonuclease (Exo) and polymerase (Pol) domains are located in the N-terminal half of this protein whereas the C-terminal half contains no experimentally characterized or readily detectable domains except for two Zn-finger modules at the end of the sequence [11,17,24-26]. The Pol ε holoenzyme heterotetramer [27], the 20 Å resolution structure of which has been determined by cryo-electron microscopy (cryo-EM) [28], contains, in addition to the large catalytic subunit, three smaller subunits, DPB2-4; the DPB2 subunit is essential for viability, and its proper structure is required for high fidelity of genome replication [29]. Site-directed mutagenesis experiments demonstrated that the Zn-fingers of Pol ε are required for its interaction with DPB2 [29]. Deletion of the other two accessory subunits is not lethal but leads to elevated mutation rates [30,31].

The sequences of the Zn fingers in Pol α, Pol δ and Pol ζ are adjacent to the C-terminal portion of the catalytic domain that is homologous to the sequences of the Thumb subdomain in the available crystal structures of B-family DdDps. By contrast, the Zn fingers in Pol ε are separated from the N-terminal catalytic domains by a large insert that is similar in size to the N-terminal Exo-Pol module. Examination of the Cryo-EM structure [28] indicates that this insert and the DPB2-binding subdomain (Zn fingers) are, largely, structured and bound to each other; furthermore, the presence of this insert places the DPB2-binding area spatially apart from the N-terminal, catalytic Exo-Pol module. Somewhat paradoxically, it was shown by deletion mutagenesis and site-directed mutagenesis that the N-terminal, catalytic portion of Pol ε is not required for viability whereas the uncharacterized C-terminal portion is essential [11,16,24-26].

We employed secondary structure prediction and fold recognition in combination with different sequence similarity search strategies in an attempt to elucidate the origin and possible functions of the essential C-terminal region of Polε. Secondary structure prediction and automated three-dimensional model building for the N-terminal 1200 amino acids of human Pol ε using the Phyre server [32], as expected, revealed a typical DNA polymerase fold (pdb: 1wn7, 1d5a, 1s5j, 1q8i, 2gv9, 2p5o) with a 100% confidence. Strikingly, the search with the remaining amino acids 1201–2286 of human Pol ε also revealed a DNA polymerase fold for the sequences preceding the Zn fingers with the confidence of 95% (*E. coli *DNA polymerase II, PDB code 1q8i), 90% (*Desulfurococcus sp. tok *DNA Polymerase, 1d5a) and 85% (*Thermococcus kodakara*ensis family B DNA polymerase, 1wn7). Although we did expect to detect some Thumb subdomain-like fold that would stabilize the positions of Zn fingers, the discovery of the entire second polymerase and exonuclease module was highly surprising. This unexpected finding prompted us to initiate a further, in-depth sequence analysis in an attempt to elucidate the origin and possible functions of the essential C-terminal region of Pol ε.

A PSI-BLAST search [33] with the C-terminal portion of the Pol ε sequence from Saccharomyces cerevisiae (amino acid positions from 1170 to 2085 aa) used as the query (with E = 0.001 inclusion threshold and composition based statistics on) reveals similarity to the sequence of DNA polymerase II of *Photobacterium profundum *(GI:90410522) of the B-family at the 3^rd ^iteration, with E-value = 2e-05; numerous sequences of B-family polymerases were detected in subsequent iterations. The same sequence was used as a query for an HHpred search [34]. This method detects the similarity with a B-family polymerase from the archaeon *Thermococcus sp*. (pdb: 1qht) with E-value = 4.9e-06 as the second top hit (the first one is a self-hit to pfam08490: DUF1744, Domain of unknown function) and several additional hits to different sequences and profiles of B-family polymerases with statistically significant E-values.

**Figure 1:**
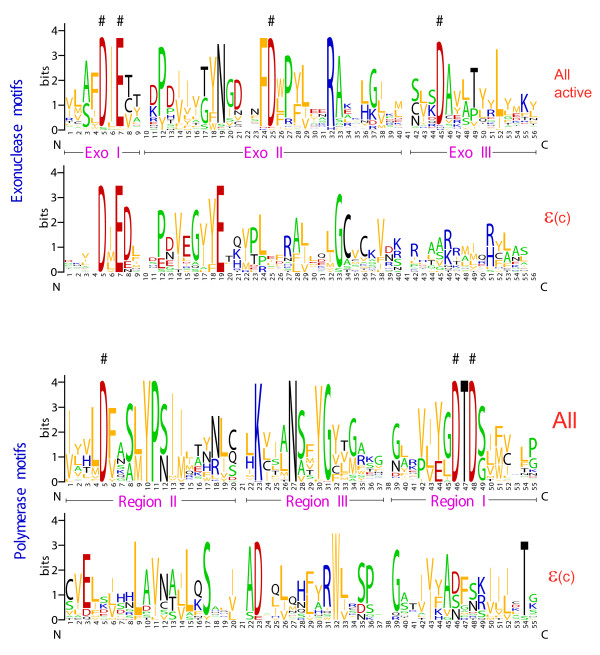
**The conserved motifs of exonuclease and polymerase catalytic domains of active B-family polymerases compared to inactivated C-terminal domains of polymerases ε**. The motifs are represented as four sequence LOGOs, from top to bottom: all active Exo domains of B-family polymerases from the alignment in Additional File 1 (archaeal, proteobacterial, and eukaryotic Polδ and N-terminal domain of Polε); inactivated C-terminal domain of Polε; all active Pol domains; inactivated C-terminal domain of Polε. The motifs that contribute to the active centers are denoted Exo I-III and Region I-III for the Exo and Pol domains, respectively, and the catalytic residues are shown by #.

The results of these searches strongly support the possibility, originally brought up by the structural comparisons described above, that the C-terminal portion of Pol ε is homologous to B family polymerases. A more detailed analysis showed that, although the C-terminal region of Pol ε readily aligned with B family DdDps, the motifs that contain the catalytic amino acid residues in both the Exo and Pol domains are disrupted in the Pol ε sequence, with the only apparent exception of the 'DIE' motif of the Exo domain (Figure 1). The partial conservation of this motif might indicate that the inactivated Exo domain of Pol ε retains metal-binding capacity, although not the catalytic activity. Thus, it appears that the C-terminal portion of the eukaryotic Pol ε is a derived B-family DdDp in which both the Exo domain and the Pol domain are inactivated. Inactivation of catalytic domains or subunits in DNA polymerase has been observed previously. In particular, we recently described a family of inactivated B family polymerases that is widespread in diverse archaea [35]. In addition, the small subunits of eukaryotic B-family DdDps including DPB2, the essential second subunit of Pol ε, are inactivated versions of the exonuclease subunits of archaeal PolD [36-40]. However, to our knowledge, Pol ε is the first detected case of the combination of an active and inactive polymerase within the same protein. Thus, it seems particularly remarkable that both essential subunits of Pol ε are inactivated derivatives of replicative enzymes. The fusion of active and inactivated B-family polymerases in Polε supports the prediction that active and inactive forms function in concert in archaeae and some bacteria although so far no fusions analogous to Polε were detected in prokaryotes [35]. As proposed previously, inactivated polymerase subunits are likely to perform essential functions in the assembly of replicative complexes [25,35,36].

**Figure 2:**
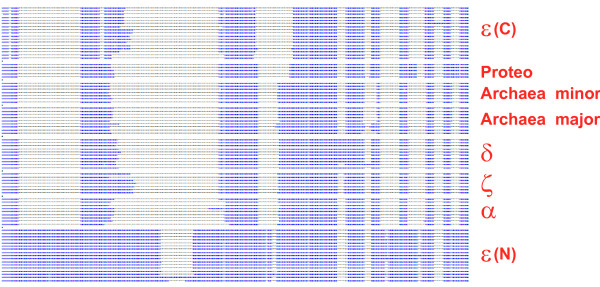
**A schematic diagram of conserved blocks and specific inserts in the most conserved part of the alignment of polymerase catalytic domain of different groups of B-family DNA polymerases**. For the actual alignment, see Additional File 1.

In general, when two homologous domains follow one another within the same protein, one would be inclined to suspect that they evolved by tandem duplication. However, the inactivated C-terminal part of Polε was much more similar to a variety of B-family polymerases, in particular, bacterial ones, than to the active, N-terminal polymerase moiety of Pol ε. Moreover, the latter, active moiety of Polε differed from other B-family DdDps including the inactivated C-terminal part of Polε by the presence of multiple, unique inserts (Figure 2). These observations do not support the intuitively plausible hypothesis of a tandem duplication in Pol ε and prompted us to investigate in greater detail the domain architectures of eukaryotic DdDps and their likely origins.

### Unexpected evolutionary affinities of the Zn-finger modules of eukaryotic B family DNA polymerases

**Figure 3:**
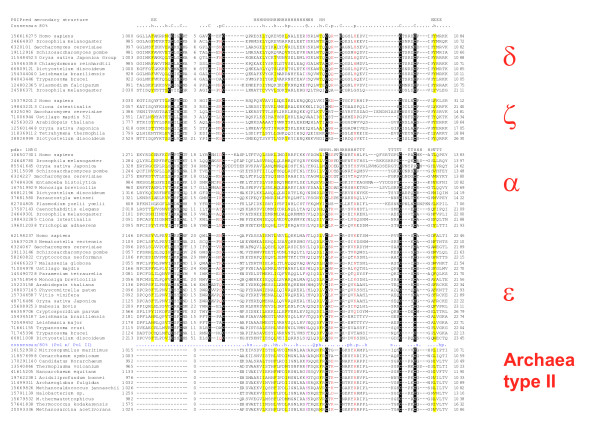
**Multiple alignment of the two-Zn-finger modules of eukaryotic Pol α, ζ, δ, and ε, and the single Zn-finger of archaeal PolD**. The sequences are denoted by their GI numbers and species names. The positions of the first and the last residues of the aligned region in the corresponding protein are indicated for each sequence. The numbers within the alignment represent poorly conserved inserts that are not shown. The cysteine residues that are essential for Zn-binding are shown by reverse shading. The coloring is based on the consensus shown underneath the alignment; 'h' indicates hydrophobic residues (ACFILMVWY), 'p' indicates polar residues (EDKRNQHTS). Additional consensus line at the top of archaeal polymerase II alignment indicates additional conservation between polymerase ε and archaeal polymerase II: 's' indicates small residues (ACDGNPSTV). The predicted secondary structure is shown above the alignment and is compared to the NMR structure that is available for human Pol α (pdb: 1N5G) [67] that is shown on top of the Pol α alignment; 'H' indicates α-helix, 'E' indicates extended conformation (β-strand) and 'T' indicates a turn.

Eukaryotic B-family DdDps contain two Zn-finger modules at their C-termini. Unexpectedly, when examining the results of PSI-BLAST searches with the C-terminal portion of Pol ε as a query, we detected significant similarity to the C-terminal Zn-finger modules of the archaeal PolD rather than to those of Pol α, Pol δ, or Pol ζ. Specifically, the PSI-BLAST search with C-terminal Zn-finger sequence of the yeast Pol ε (amino acids 2116 to 2199) reveals highly significant similarity to the C-terminal Zn-fingers of many archaeal Pol D sequences (E-value 4e-06 in the second search iteration). By contrast, significant similarity to the Zn-fingers of other eukaryotic B-family polymerases could not be easily demonstrated. The reverse search with the Zn-fingers of archaeal PolD yielded, essentially, the same results (data not shown). The same relationship was detected using HHpred: the Zn-finger sequence from yeast Pol ε gave the top hits to several profiles of PolD with E-value as low as 1.4e-24. The multiple alignment of the Zn-finger domains of DdDps clearly reveal the specific similarity between the distal Zn-finger of Pol ε and the sole Zn-finger of the archaeal Pol D as opposed to the limited similarity to the Zn-fingers of other eukaryotic B-family polymerases (Figure 3). These observations suggest an unexpectedly complex evolutionary scenario for the origin of eukaryotic DdDps from archaeal ancestors. After performing this analysis, we became aware of the fact that the specific similarity between the Zn-finger of the catalytic subunit of Pol ε and archaeal PolD has been noticed previously although evolutionary implications of this finding have not been examined [40].

### Origin of eukaryotic B-family DNA polymerases

**Figure 4:**
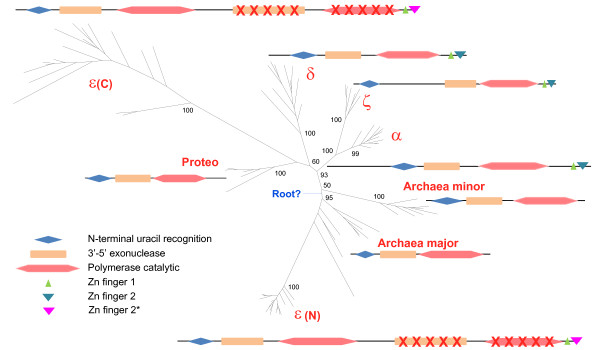
**Unrooted phylogenetic tree of B-family DNA polymerases**. The tree was constructed using the conserved blocks from the Exo-Pol alignment (see Additional File 1). The tree is rendered as a scheme, with only the major groups denoted; for the complete tree, with all species indicated, and trees constructed with alternative methods, see Additional File 2. The tree is overlaid with schematics of domain architectures which are given for representatives of each group (*Saccharomyces cerevisiae *sequences for polymerases α, ζ, δ, ε and those from *Sulfolobus solfataricus*, *Pyrococcus furiosus *(pdb:2JGU), and *Escherichia coli *(pdb:1Q8I) for archaeal minor, archaeal major and proteobacterial groups, respectively). The domains are shown roughly to scale. Inactivated C-terminal domains of polymerases ε are crossed. Dashed line indicates the portion of the sequences corresponding the adjacent tree branch. Zn-finger 2* denotes the distinct version of this module in Pole that is highly similar to the Zn-finger of archaeal PolD (see text for details). The proposed position of the root is shown by an arrow.

Because of the high sequence divergence of the Pol ε C-terminal domain, we were able to construct a reliable alignment only for approximately 280 amino acid residues from the Exo and Pol domains of B-family DdDps. Nevertheless, when this alignment was employed for phylogenetic tree reconstruction, the topology of the tree was quite stable as demonstrated with a variety of tree-building methods and different parameter combinations (Figure 4; see Methods for details). The results were, mostly, compatible with those of previously published phylogenetic analyses of B-family DNA polymerases [35,41,42]. The tree has a complex structure, with the active, N-terminal region of Pol ε clustered with the "major" group of archaeal B-family polymerases (PolBI) that is represented in nearly all archaea, whereas the rest of the eukaryotic B-family polymerases including the inactivated C-terminal portion of Pol ε are affiliated with a distinct, "minor" group of polymerases (PolBII) found in a smaller subset of archaea (Figure 4). These findings are in a general agreement with the previous results of phylogenetic analysis of archaeal and eukaryotic B-family polymerases [43,44].

**Figure 5:**
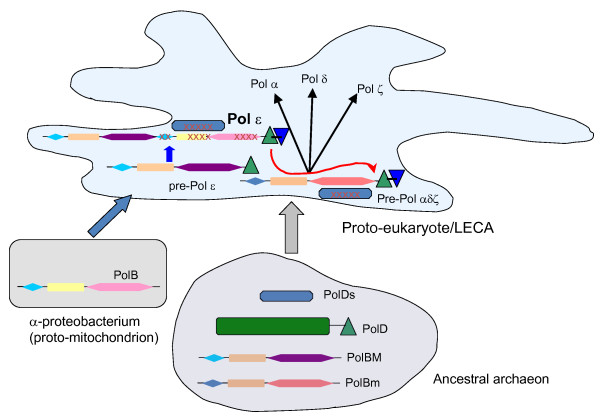
**A putative evolutionary scenario for the origin of eukaryotic B-family DNA polymerases from prokaryotic ancestral forms**. The scheme is rendered within the framework of the symbiotic scenario of the origin of eukaryotes whereby the symbiosis of an archaeon with an α-proteobacterium gave rise to the mitochondrion and triggered eukaryogenesis. The domains are designated by unique shapes as in Figure 4. PolBM, the "major" form of archaeal B-family DNA polymerase (PolBI [43]); PolBm, "minor" form of archaeal B-family DNA polymerase (PolBII [43]; PolDs, small subunit of archael PolD (active exonuclease). Inactivation of PolDs in the protoeukaryote (the Last Eukaryotic Common Ancestor, LECA) is denoted by crosses. The origin of Pol ε is depicted as insertion of a bacterial B-family polymerase between the catalytically active module derived from the archaeal PolB-M and the Zn-finger derived from the archaeal PolD.

Together with the observations on the Zn-finger domains of archaeal and eukaryotic B-family polymerases, the results of phylogenetic analysis suggest an unexpectedly complicated scenario of the evolution of eukaryotic DdDps that is not limited to duplications and diversification as central trends at the early stage of eukaryogenesis [45]. Instead, the results suggest distinct archaeal pedigrees for eukaryotic polymerases and imply that the archaeal ancestor of eukaryotes possessed at least two B-family polymerases as well as PolD from which the "major" B-family form acquired the Zn-finger, either prior to or during eukaryogenesis (Figure 5). The combination of a B-family polymerase with the PolD Zn-finger is not seen in any of the sequenced archaeal genomes, in accord with the conclusions of a recent phylogenetic analysis that derives the "archaeal" subset of eukaryotic genes from a deep branch of archaea [46]. Under this scenario, eukaryotic Pol ε and the rest of the eukaryotic B-family polymerases appear not to be ancient eukaryotic paralogs *sensu strictu*, but rather, pseudoparalogs originating from paralogous archaeal ancestors [45].

The subsequent events in the evolution of eukaryotic B-family DdDps that occurred prior to the radiation of the major lineages of eukaryotes included not only two duplications of the Pol-Exo block that led to the origin of polymerases α,δ, and ζ, but also the duplication of the Zn-finger, probably, in the ancestral Polε, with the subsequent acquisition of the two-finger module by the common ancestor of Polα, Polδ, and Polζ. The inactivated C-terminal portion of the Polε is more likely to result from a fusion of two distantly related B-family polymerases as opposed to the intragenic duplication scenario. The topology of the phylogenetic tree suggests that the source of the C-terminal portion of Pol ε could be a proteobacterial (or bacteriophage) B-family polymerase (Figure 4) although, given the long Pol ε branch, its origin cannot be determined with any confidence. In principle, a long-branch artifact could even obscure a duplication of the N-terminal portion of Pol ε; however, this seems unlikely considering that the N-terminal sequence shows a distinct pattern of indels as opposed to a common pattern in the C-terminal sequence and the rest of the eukaryotic B-family polymerases (Figure 2).

## Conclusion

The analysis described here reveals the complexity of the evolution of only one, although biologically central, group of eukaryotic proteins, the B-family DNA polymerases involved in genome replication and some repair processes. Evolution of the eukaryotic B-family polymerases seems to have involved several previously unnoticed events. At face value, eukaryotic B-family DdDps appear to be chimeric with respect to their archaeal ancestors, with the catalytic portion (Pol and Exo domains along with the N-terminal uracil-binding domain [47]) derived from archaeal B-family polymerases and the Zn-finger derived from PolD (Figure 5). The derivation of the small subunits of eukaryotic B-family polymerases, such as DPB2, from the exonuclease subunits of the archaeal PolD further emphasizes the joint contributions of the B-family and D-family archaeal polymerases to the evolution of the eukaryotic replication machinery. It is unclear, however, at what stage of evolution the chimeric polymerases evolved. The possibility remains that this fusion of domains that, in archaea, so far have been detected separately, is characteristic of the hypothetical (extinct or extant but not yet discovered) deep lineage of archaea that provided the archaeal heritage of eukaryotes [46].

The unexpected observation that triggered this analysis is the presence, in the C-terminal regions of the large, catalytic subunits of all eukaryotic Polε, of apparently inactivated versions of the Exo and Pol domains. These sequences are conserved in all eukaryotes and, notably, have been identified as essential by deletion mutagenesis [11,25,26]. Thus, it appears certain that, despite the inactivation of both catalytic activities, the C-terminal portion of Pol ε plays a key role in DNA replication of all eukaryotes, conceivably, as a structural component that is indispensable for the assembly of replication complexes at the origins [48], with likely additional functions in repair and cell cycle regulation [16]. Inactivation of enzymatic activities of polymerase subunits is becoming a rather general theme in the evolution of the architecture of the replication machinery, two other cases being the inactivation of the nuclease domain in the small subunits of eukaryotic B-family polymerases [36,40,49], and the inactivation of both catalytic domains in a distinct family of archaeal polymerase homologs [35]. Strikingly, the evolution of Pol ε seems to have involved a concerted inactivation of both the Exo and Pol domains of a B-family polymerase (possibly, one that fused with the ancestral B-family polymerase) and of the exonuclease subunit of PolD, suggesting that selective pressure exists for the utilization of these inactivated derivatives of replicative enzymes as structural components of replicative complexes.

Another case of functional inactivation despite structural conservation is the uracil recognition domain that is conserved in archaeal and eukaryotic B-family polymerases (Fig 4) but lost the capacity to sense uracil in front of the moving polymerase in eukaryotes [50]. Mechanistic characterization of the inactivated polymerase subunits and domains is expected to shed new light on the functions of the replication apparatus.

On a more general note, the present analysis indicates that footprints of undetected evolutionary events with important functional implications are still lurking in even supposedly well-characterized proteins. Conceivably, a variety of non-trivial evolutionary connections between eukaryotic proteins and their prokaryotic ancestors remain to be discovered, leading to unusual evolutionary scenarios.

## Methods

All analyzed sequence were from the NCBI's RefSeq database [51]. Multiple alignments of protein sequences were constructed by combining the results obtained with the PROMALS program [52] and the MUSCLE program [53], followed by a minimal manual correction on the basis of local alignments obtained using PSI-BLAST (see Additional File 1). Protein sequence motifs were represented using sequence LOGOs where the height of the amino acid symbols is a function of the frequency of the given amino acid in the given position [54,55]. Protein secondary structure was predicted using the PSIPRED program [56]. Protein fold recognition was performed using the Phyre server [32].

Maximum likelihood (ML) phylogenetic trees were constructed from the alignment of the most conserved positions of the Pol and Exo domains of the B-family polymerases (279 positions altogether, with only a few gaps within the conserved blocks) by using the MOLPHY program [57,58] with the JTT substitution matrix to perform local rearrangement of an original Fitch tree [59]. The MOLPHY program was also used to compute RELL bootstrap values. The topology of the tree was validated using independent ML methods implemented in the Treefinder [60] and RaxML [61] programs with optimized JTT, WAG and RtRev substitution matrices (see Additional File 2).

## Competing interests

The authors declare that they have no competing interests.

## Authors' contributions

THT identified the second nonfunctional polymerase and exonuclease domains of Pol ε using secondary structure and protein fold predictions and incepted the study; KSM contributed to sequence analysis, performed the phylogenetic analysis, and wrote an original draft of the manuscript; IBR and YIP contributed to sequence analysis and interpretation of the results; EVK contributed to sequence analysis and interpretation of the results, and wrote the final manuscript; all authors read, edited, and approved the final manuscript.

## Reviewers' reports

### Reviewer 1: Patrick Forterre, Institut Pasteur

The paper by Tahirov and colleagues reports a very exciting observation: they have shown convincingly, using a combination of *in silico *approaches based on structural comparison and iterative Psi-BLAST analyses, that the C terminal domain of the eukaryotic DNA polymerase ε, corresponds to an inactivated DNA polymerase of the B family. The eukaryotic DNA polymerase ε thus appears to be formed by the fusion of an active DNA polymerase B (in N-terminal) and an inactive DNA polymerase B (in C-terminal). Amazingly, the inactive DNA polymerase B does not seem to have originated from a duplication of the active one, but by the fusion of a bacterial-like DNA polymerase B (such as *E. coli *DNA polymerase II). This is a very interesting observation that deserves publication. The authors also notice that the two Zinc fingers of the eukaryotic DNA polymerase ε are more related to Zinc fingers of archaeal DNA polymerases D than to those of other DNA polymerases B. This is in line with the fact that archaeal DNA polymerases D and eukaryotic DNA polymerases ε both interact with homologous subunits in Archaea and Eukarya. From these two observations, the authors speculate about the origin and evolution of eukaryotic DNA polymerases. I think that the authors should more clearly distinguish between their observations and evolutionary hypotheses. For instance, in the abstract, the hypotheses are described in the "result section" and even introduce this section as if they were *bona fide *results. The main and exciting result is only presented as an additional observation!!! "*In addition, we found that*.....". I think that the hypothesis favoured by the author should be mentioned only in the conclusion.

Authors response: *We appreciate these constructive suggestions and have revised the Abstract accordingly. In the main text, the description of the inactivated module of Pol ε already preceded the rest of the analysis, so no change was necessary*.

Ideally, the authors should have discussed their observations in the context of alternative hypotheses on the origin of eukaryotes and the eukaryotic DNA replication apparatus (see below). In my opinion, in discussing evolutionary scenarios, terms such as "archaeal ancestor" (already in the title and abstract conclusions) (see Figure also 5) should be avoided. The term archaeal ancestor is confusing since the common ancestor of Archaea and proto-eukaryotes was probably neither a proto-eukaryote nor an archaeon. Similarly, the Human does not descend from Apes, but Apes and Human have a common ancestor.

Authors response: *This point is often brought up, and a reminder, we hope, will be helpful to the reader. It is true that Homo sapiens did not evolve from Pan troglodytes or any other living great ape species but rather shares a common ancestor with them. However, that common ancestor was, necessarily, an ape (distinct from any extant ape, of course), so the phrase "ape ancestor of humans" is not confusing, in our opinion. Ditto regarding "archaeal ancestor of eukaryotes"*.

From our own analysis of the evolution of the DNA replication apparatus (unpublished), it is indeed likely that the last common ancestor of Archaea had two DNA polymerases of the B family and one of the D family (as suggested in Figure 5A) and this was possibly also the case for the last common ancestor of Archaea and proto-eucaryotes (as suggested by the authors). The authors imagine a scenario of evolution going from this "simple" ancestor to modern eukaryotes (transformation of the two ancestral polymerases B in four polymerases B and loss of the polymerase D in the lineage of modern eukaryotes). However, one cannot exclude other scenarios, such as the presence of more than two polymerases B in the common ancestor of archaea and proto-eucaryotes (with loss of DNA polymerase D in Archaea and of some DNA polymerase B in Archaea), and/or introduction of DNA polymerases of viral origin in Archaea and/or in the lineage of proto-eucaryotes [62]. Since viral DNA polymerases of the B family are intermixed with cellular DNA polymerases in phylogenetioc tree [63,64], it should be in any case interesting to extend the present analysis to viral DNA polymerases as well.

Authors response: *We agree that alternative scenarios are imaginable. They might somewhat less parsimonious but parsimony is at best a rough guide in the study of such complex evolutionary scenarios. Analysis of viral polymerases is interesting although it is complicated by the typical high rate of evolution of viral proteins, even essential ones*.

### Reviewer 2: Arcady Mushegian, Stowers Institute

The authors discuss the compelling evidence for the complex evolutionary history of eukaryotic Family B DNA polymerases. Observations and their analysis are technically sound, and I have only minor questions.

1. Abstract: "of archaeal, eukaryotic, and bacterial B-family DNA polymerases, the main replicative polymerases in archaea and eukaryotes" is awkward.

Authors' response: *corrected to a (hopefully) less awkward phrase*

2. Ibid. "eukaryotic B-family polymerases, most likely, originate from two distinct archaeal ancestors" – perhaps change to "there are two subgroups of eukaryotic B-family polymerases, each most likely originating from its own archaeal B-family ancestor". Otherwise. "two distinct archaeal ancestors" can be mistaken for the description of B+D chimera in the following sentence.

Authors' response: *modified for clarity*

3. "As proposed previously, inactivated polymerase subunits are likely to perform essential functions in the assembly of replicative complexes" (also Conclusions) – a bolder suggestion may be that these proteins still facilitate a subset of catalytic reactions, if the maintainance of a proper conformation of subsrates/ligands is sufficient for catalysis – processive synthesis may not work well that way, but perhaps some sort of proofreading or ejection of abortive products might – discuss?

Authors' response: *a bold proposal, indeed, in our opinion, too bold to be considered justified at this time. Actually, it has been shown that the 145 kDa proteolytic fragment of Pol ε, missing the C-terminal Pol Exo module, is indistinguishable from the four-subunit complex with respect to the exonuclease and polymerase activities but less rapidly dissociates from primer-template *[65]*It is also known that the C-terminal domain of Pol2p and/or the auxiliary subunits are specifically involved in dsDNA-binding *[66]. *Obviously, the C-terminal domain of Pol ε is critically important for replication but the complete elucidation of its specific functions requires much more experimentation. Nevertheless, in the revised version of the manuscript we are more specific about the possible role of the conserved DIE motif of the inactivated module of Pol ε*.

4. Evolutionary scenario and Fig. 5: Why proteobacterial-type PolB, the source of the C-terminal domain tandem in eukaryotic Pol epsilon, has to be the symbiogenetic/mitochondrial acquisition – can it be a phage contribution instead?

Authors' response: *in principle, it could be a phage contribution but we do not see any specific indications of such an origin of the inactivated polymerase module of Pol ε*.

### Reviewer 3: Chris Ponting, Oxford University

This manuscript reports the identification of a tandem exonuclease-polymerase homology module within the C-terminal regions of DNA polymerase epsilons. The authors propose these domains arose by gene fusion rather than intra-gene duplication and that they dispensed with their enzymatic activities. Also discussed are the evolutionary implications of similarities between zinc fingers of DNA polymerase epsilon and archaeal PolD.

This is a well-written and compelling report that contributes significantly to our understanding of the evolution of cellular DNA replication and repair. The sequence similarity methods and the statistical analyses used are entirely appropriate. These findings should now re-focus attention on the molecular mechanisms of these apparently inactivated domains in Pol-epsilon.

Authors' response: *We appreciate these constructive comments and cannot agree more with regard to the importance of experimental investigation of the functions and mechanisms of the inactivated module of Pol ε. Moreover, such experiments are currently underway in the laboratory of one of us (YIP) at the University of Nebraska Medical Center*.

## Supplementary Material

Additional file 1 **Multiple alignment of Exo-Pol modules of B-family DdDps (text).** Click here for file

Additional file 2 **Phylogenetic trees of B-family DdDps (in Newick format) constructed with different methods (text).** Click here for file
